# Poorly Differentiated Extraocular Sebaceous Carcinoma Masquerading as an Abscess

**DOI:** 10.7759/cureus.87789

**Published:** 2025-07-12

**Authors:** Eleanor Tung-Hahn, Alina Yang, Manojkumar T Patel, Jonathan Zager

**Affiliations:** 1 College of Osteopathic Medicine, Lake Erie College of Osteopathic Medicine, Bradenton, USA; 2 Biological Sciences, University of Maryland, College Park, USA; 3 Dermatopathology, DermPath Diagnostics, Tampa, USA; 4 Surgery, Moffitt Cancer Center, Tampa, USA

**Keywords:** extraocular sebaceous carcinoma, poorly differentiated sebaceous carcinoma, sentinel lymph node biopsy (slnb), treatment, wide local excision

## Abstract

Extraocular sebaceous carcinoma (SC) is an uncommon but potentially aggressive tumor. Typical presentation is a red-yellow nodule, often ulcerated, on the head and neck of an older individual. Factors increasing the risk of SC include genetic mutations, ultraviolet light exposure, prior radiation, and immunosuppression. A 63-year-old Caucasian man presented with an enlarging, painful subcutaneous mass on his left arm, which was ultimately diagnosed as a poorly differentiated extraocular SC. Given the aggressive tumor subtype and large size of the lesion, he was definitively treated with wide local excision and sentinel lymph node biopsy. While his past medical history and family history were significant for colon cancer, he declined further genetic workup and testing. Risk factors for metastasis of SC include a tumor diameter greater than 2 cm, rapid growth, high histologic grade, or poor differentiation. As poorly differentiated extraocular SC is associated with a higher rate of nodal involvement, management with complete surgical resection and evaluation (sentinel lymph node biopsy or imaging) to determine whether metastatic spread has occurred may be of benefit to establish if further procedures, radiation, or therapeutics are indicated.

## Introduction

Extraocular sebaceous carcinoma (ESC) is a rare but potentially aggressive malignancy. It mainly occurs in older adults on the head and neck, often presenting as a solitary, painless, red-yellow, ulcerated nodule [1]. Definitive diagnosis is made based on histologic and immunohistochemical analysis [1,2]. We report a unique clinical manifestation of a large, poorly differentiated ESC on the arm and present a management approach consisting of surgical resection and sentinel lymph node biopsy (SLNB). Ascertainment of metastatic spread in high-grade histology sebaceous carcinomas (SCs) can guide whether further intervention, including lymph node dissection, radiation, or other therapy, is needed [3-5].

This article was previously presented as a meeting poster at the Florida Academy of Dermatology annual meeting in Palm Beach, FL, on June 28, 2024.

## Case presentation

**Figure 1:**
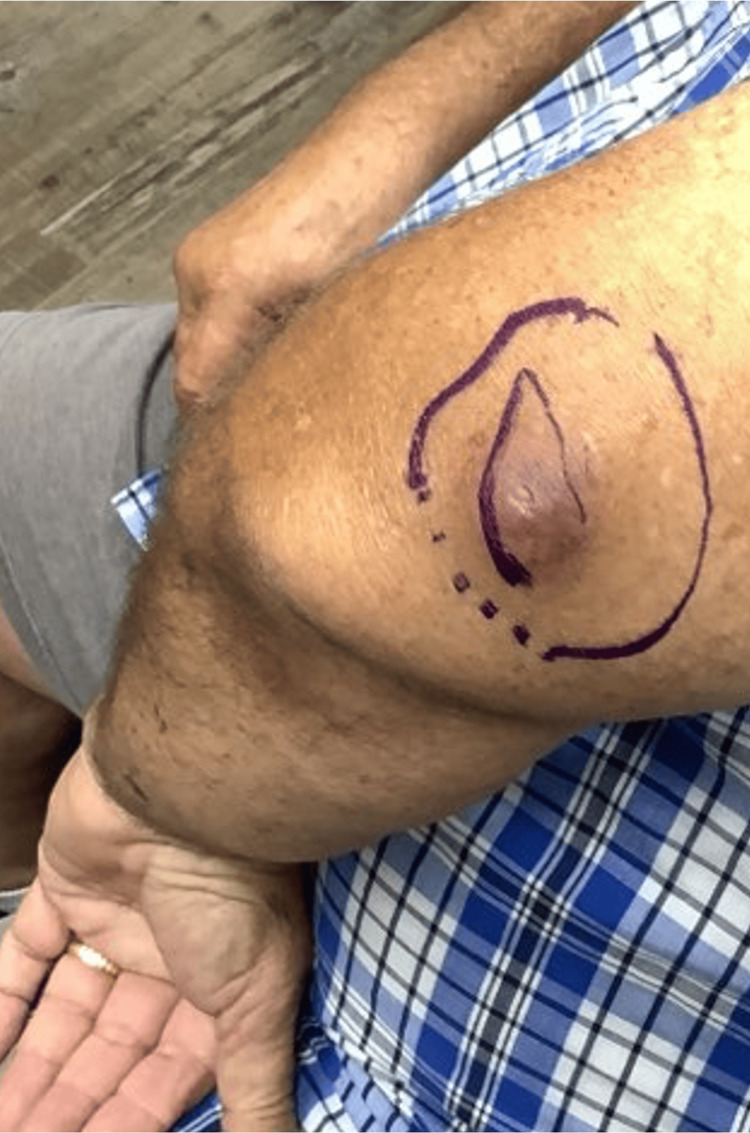
Tender subcutaneous mass on the left upper arm.

**Figure 2:**
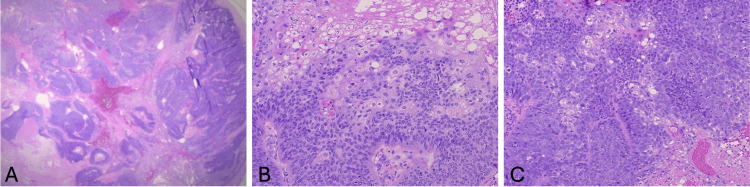
Low-resolution (A) high-resolution (B and C) histology. (A-C) The tumor is mainly composed of lobules of atypical basaloid cells with minimal differentiation toward multivacuolated cells. Mitotic figures are present. Diagnosis: poorly differentiated sebaceous carcinoma.

**Figure 3:**
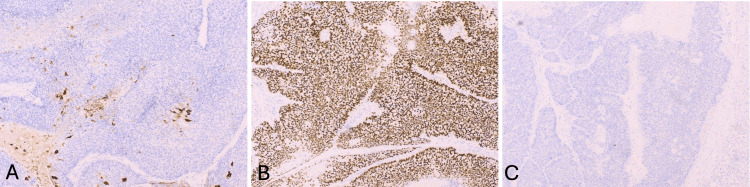
(A-C) Immunohistochemical stains of tumor cells. (A) EMA is focally positive. (B) Androgen receptor is positive. (C) BerEp4 is negative.

A 63-year-old male (Fitzpatrick skin type II) presented with a two-year history of a progressively symptomatic, enlarging, tender, subcutaneous 2.5 × 2.5 cm nodule on his left upper arm. The lesion was initially presumed to be an abscess and was excised for definitive diagnosis after it failed to resolve with incision and drainage and oral doxycycline. At excision, the lesion appeared as a painful 7 × 6 cm mass (Figure 1). This initial excision revealed an ulcerated, poorly differentiated SC. Microscopic examination demonstrated a proliferation of atypical basaloid cells (Figures 2A-2C). Within these basaloid nests were poorly differentiated multivacuolated cells. On immunohistochemistry, tumor cells were androgen receptor positive, and epithelial membrane antigen (EMA) was focally positive (Figures 3A, 3B). Antihuman epithelial antigen (Ber-EP4) staining was negative (Figure 3C). While the bulk of the lesion appeared to be excised, it focally involved the peripheral margins. Upon further query, the patient noted that he had surgery for colon cancer at age 25 and had a family history significant for internal malignancies (colon cancer in his mother and father and pancreatic cancer in his sister). His occupation was a long-distance truck driver. He had no history of radiation, immunosuppression, skin cancer, or sebaceous tumors. He was married and had no children. Given the aggressive features of his tumor, past history of colon cancer at a young age, and family history of colon and pancreatic cancers, he was referred to Moffitt Cancer Center for further treatment. He underwent radical resection under sedation with 1 cm margins to fascia and SLNB. Margins were clear, and SLNB of the left axillary node was negative. He declined further genetic testing and imaging at that time. He was recommended to follow up every six months for monitoring.

## Discussion

ESC typically presents as a painless pink or yellow nodule that is often ulcerated on the head and neck. Rarer sites include the trunk, extremities, and genitalia [1]. From recent systematic reviews, the peak age for presentation was 60-70 years old, and 58% of ESCs occurred in men. The mean tumor diameter was 1-4 cm. Most ESCs were present for 1-2 years before diagnosis [6,7]. ESC can be associated with Muir-Torre syndrome (MTS), a variant of Lynch syndrome, characterized by sebaceous neoplasms or keratoacanthomas and visceral malignancy. MTS has characteristic mutations in mismatch repair genes. The genes most commonly mutated in MTS are *MSH2*, *MLH1*, *MSH6*, and *PMS2* [8]. Typically, MTS patients frequently develop ESC at a younger age, may have multiple sebaceous neoplasms, and often have ESCs located outside of the head and neck anatomic regions. Cutaneous lesions occur before or concurrently with visceral cancers in 40% of MTS cases [1]. Factors increasing the risk of ESC include genetic mutations (MTS), ultraviolet light exposure, prior radiation, and immunosuppression [9]. Histologically, SC appears as sheets or lobules of atypical basaloid cells with varying degrees of sebaceous differentiation separated by fibrovascular stroma with infiltration into surrounding tissues. Well-differentiated tumors have higher proportions of sebocytes, whereas poorly differentiated SCs have greater proportions of undifferentiated basaloid cells with more pleomorphism, atypia, mitoses, and necrosis [2].

Immunohistochemical staining can help definitively define the diagnosis of ESC. A large study noted positive immunohistochemical markers in ESC included nuclear factor XIIIa (AC-1A1), EMA, cytokeratin AE1 and AE3, androgen receptor, adipophilin, and perilipin. Mostly negative markers included carcinoembryonic antigen, S100, HMB45, SOX10, CD5, GCDFP-15, D2-40, and Ber-EP4 [7]. In poorly differentiated SC, one study found androgen receptor positivity to be a more sensitive and reliable marker than EMA [10].

Utilization of immunohistochemistry can help distinguish SC from other diagnoses, such as basal cell carcinoma with sebaceous differentiation, squamous cell carcinoma, melanoma, metastatic renal carcinoma, and clear cell sarcoma [1,9]. Summarized findings on immunohistochemistry to differentiate between ESC, basal cell carcinoma, and squamous cell carcinoma can be found in Table 1 [2,7].

**Table 1:** Summary of immunohistochemical staining to differentiate between sebaceous carcinoma/extraocular sebaceous carcinoma, basal cell carcinoma, and squamous cell carcinoma. ++ = positive almost always; + = usually positive; +/- = occasionally positive; -- = almost never positive

Malignancy	EMA	Androgen receptor	Factor XIIIa	Adipophilin	Ber-EP4	CK7
Sebaceous carcinoma	++	+	+ (particularly extraocular sebaceous carcinoma)	++	--	++
Basal cell carcinoma	--	- (rarely +)	--	--	++	+/-
Squamous cell carcinoma	++	--	--	--	--	- (rarely +)

Risk factors for regional and distant metastasis of SC include a primary tumor diameter greater than 2 cm, rapid growth, high histologic grade or poor differentiation, and a higher number of Mohs stages if the procedure is done [3,11]. Prognosis in patients with ESC is relatively poor, as assessed in a review of the Surveillance, Epidemiology, and End Results database (2000-2012). Patients had a 10-year survival rate of 57% and a five-year survival rate of 78% [6]. Male sex, Black race, and extraocular site were associated with higher all-cause mortality [6]. A smaller, single-site study found the ESC five-year survival rate to be better at 97% [11]. Based on a review of 3,211 SCs from the National Cancer Database (NCDB) (2004-2016), ESCs on the trunk and extremities were more likely to be larger, well-differentiated, and have fewer nodal metastases. However, poorly differentiated ESCs were associated with a higher rate of nodal metastasis [12]. In a retrospective study of cutaneous adnexal malignancies, including ESC, overall tumor size (>2 cm) and presence of nodal metastatic disease, as demonstrated by SLNB, had a significant negative impact on overall survival [4]. Nodal status also had an impact on recurrence, suggesting the potential utility of SLNB [4]. In another NCDB survey of 1,388 SCs, researchers found 4.9% of cases had metastases. Of the 149 SLNBs performed, 7% were positive. The rate of SLNB positivity was only slightly less for ESC versus periocular SC. Approximately two-thirds of positive SLNBs were found to be high histologic grade SC [5]. High-grade histology (poorly differentiated or undifferentiated tumors) was identified as an independent predictor of poor overall survival. Factors associated with high-grade SC included periocular site, large tumor size (>2 cm), older age, and more advanced disease. Regardless of anatomic site, patients with high-grade SC may benefit from radiographic staging and/or SLNB [3,5]. The mainstay of treatment for ESC is complete resection with preservation of function.

Guidelines for the treatment of ESC recommend margin-controlled therapies with fresh or permanent sections (Mohs micrographic surgery or complete circumferential peripheral and deep margin assessment) when feasible, or, alternately, wide local excision with margins of 1 cm radially with resection to the deep fascial plane [7]. In higher-risk ESC (large size or high-grade histology), SLNB is indicated [3-5]. If positive nodal involvement is found, further lymph node dissection and radiation may be of therapeutic benefit [7]. In the case of a patient with ESC who is not a surgical candidate, radiation can be utilized. If SC is potentially part of a syndrome, referral for further genetic testing can be recommended [1,3-7,9,12].

## Conclusions

Our case highlights the potential benefit of additional procedures, i.e., SLNB, imaging, and/or genetic testing, to rule out metastatic spread in large (>2 cm), histologically aggressive (poorly differentiated) ESCs. Poorly differentiated, large ESC can be a very aggressive cutaneous malignancy. Surgical management and workup should be tailored to the individual patient. Close clinical monitoring with multidisciplinary care can be undertaken to assess for any signs of recurrence.
